# Discriminating Paradoxical and Psychophysiological Insomnia Based on Structural and Functional Brain Images: A Preliminary Machine Learning Study

**DOI:** 10.3390/brainsci13040672

**Published:** 2023-04-17

**Authors:** Mortaza Afshani, Ahmad Mahmoudi-Aznaveh, Khadijeh Noori, Masoumeh Rostampour, Mojtaba Zarei, Kai Spiegelhalder, Habibolah Khazaie, Masoud Tahmasian

**Affiliations:** 1 Institute of Medical Science and Technology, Shahid Beheshti University, Tehran 1983969411, Iran; mortaza.afshani@gmail.com (M.A.); mzarei@me.com (M.Z.); 2 Cyberspace Research Institute, Shahid Beheshti University, Tehran 1983969411, Iran; mahmoudi.a@gmail.com; 3 Sleep Disorders Research Center, Kermanshah University of Medical Sciences, Kermanshah 6715847141, Iran; kh.noori89@gmail.com (K.N.); rostampour.ma@gmail.com (M.R.); hakhazaie@gmail.com (H.K.); 4 Department of Neurology, Odense University Hospital, 5000 Odense, Denmark; 5 Department of Clinical Research, University of Southern Denmark, 5000 Odense, Denmark; 6 Department of Psychiatry and Psychotherapy, Medical Centre—University of Freiburg, Faculty of Medicine, University of Freiburg, 79085 Freiburg, Germany; kai.spiegelhalder@uniklinik-freiburg.de; 7 Institute of Neuroscience and Medicine, Brain & Behaviour (INM-7), Research Centre Jülich, 52428 Jülich, Germany; 8 Institute of Systems Neuroscience, Medical Faculty, Heinrich Heine University Düsseldorf, 40225 Düsseldorf, Germany

**Keywords:** insomnia disorder, paradoxical insomnia, psychophysiological insomnia, classification, machine learning, multimodal imaging

## Abstract

Insomnia disorder (ID) is a prevalent mental illness. Several behavioral and neuroimaging studies suggested that ID is a heterogenous condition with various subtypes. However, neurobiological alterations in different subtypes of ID are poorly understood. We aimed to assess whether unimodal and multimodal whole-brain neuroimaging measurements can discriminate two commonly described ID subtypes (i.e., paradoxical and psychophysiological insomnia) from each other and healthy subjects. We obtained T1-weighted images and resting-state fMRI from 34 patients with ID and 48 healthy controls. The outcome measures were grey matter volume, cortical thickness, amplitude of low-frequency fluctuation, degree centrality, and regional homogeneity. Subsequently, we applied support vector machines to classify subjects via unimodal and multimodal measures. The results of the multimodal classification were superior to those of unimodal approaches, i.e., we achieved 81% accuracy in separating psychophysiological vs. control, 87% for paradoxical vs. control, and 89% for paradoxical vs. psychophysiological insomnia. This preliminary study provides evidence that structural and functional brain data can help to distinguish two common subtypes of ID from each other and healthy subjects. These initial findings may stimulate further research to identify the underlying mechanism of each subtype and develop personalized treatments for ID in the future.

## 1. Introduction

Subtyping insomnia disorder (ID) is an ongoing debate in sleep medicine and has changed over different versions of the International Classification of Sleep Disorders (ICSD) [1,2]. In ICSD-2, several ID subtypes were introduced, including paradoxical insomnia (PDI) and psychophysiological insomnia (PPI). ICSD-2 defines PDI as subjective complaints of insomnia, while polysomnography (PSG) shows near-normal sleep patterns [3]. Conversely, PPI is characterized by increased arousal before or during sleep in a routine bedroom setting. Moreover, recent studies have suggested that various ID subtypes exist with different etiology and distinct pathophysiology [4,5,6,7]. However, although ICSD-3 has emphasized the existence of the ID subtypes, it proposed to consider it as a single category, entitled chronic ID, as there are no customized therapeutic approaches for each subtype [1].

After decades of noticeable progress in brain imaging techniques, several studies aimed to explore the pathophysiology of ID using neuroimaging. Moreover, several studies have found different structural and functional brain alterations between ID patients and healthy subjects [2,8,9]. However, in recent neuroimaging meta-analysis studies, we found a lack of consistent regional abnormality in ID, probably due to various sample sizes (between 7 and 59 patients with ID across studies), different data acquisition, preprocessing, and analysis pipelines of the included studies, as well as heterogeneous clinical populations in terms of severity, duration, and subtype of ID [8,10]. Multiple ID subtypes can contribute to ID heterogeneity and ineffective treatment of ID [7,11], which could be a reason for observing inconsistent brain abnormalities across ID patients [8,10]. Moreover, subtyping is essential for parsing heterogeneity and selecting personalized treatments for each subject [12,13].

Recently, a growing trend in neuroimaging studies has been raised to decode complex and non-linearity relations between brain regions in neuropsychiatric disorders using machine learning [14] approaches. Analyzing complex and non-linear patterns in the brain at the subject level instead of the group level is a strong advantage of ML in addressing future challenges in precision psychiatry [15]. By integrating advanced computational models in psychiatry—such as ML, which is rapidly expanding in the field of neuroimaging—precision psychiatry seeks to narrow the gap between discovery and clinical application, gain a better understanding of the brain, and identify dysfunction hubs more precisely at the individual level [13]. Moreover, recent studies have utilized different brain measures to study ID and get diverse results [5,10,16,17]. This diversity might be due to the complex nature of ID that single measures cannot contain whole neurobiological alterations. Due to the widespread and various effects of ID on the brain’s structure and function, we combined different imaging modalities to reflect the global and local alteration in the brain. Moreover, using unimodal and multimodal neuroimaging with machine learning (ML) models, we aimed to determine whether the two ID subtypes, PDI and PPI, can be separated from each other and healthy controls (HC). Thus, we obtained structural features (volumetric data, i.e., grey matter volume (GMV) and cortical thickness) and functional features (low-frequency fluctuations (ALFF), degree centrality (DC), and regional homogeneity (ReHo)) for each subject as input features. We applied support vector machines to the whole-brain structural and functional matrices to classify PDI, PPI, and HC groups.

## 2. Materials and Methods

### 2.1. Subjects

In this study, 98 individuals were recruited. ID patients were recruited from the Sleep Disorders Research Center, Farabi Hospital, Kermanshah University of Medical Sciences. HCs were recruited by a local advertisement and included individuals without any history of neurological and psychiatric disorders with good sleep quality (total Pittsburgh Sleep Quality Index (PSQI) scores < 5). Exclusion criteria for all participants included current pregnancy, history of other neurological and psychiatric disorders, current use of psychiatric medications, contraindications of MRI imaging such as metallic implants or claustrophobia, substance abuse, as well as traumatic brain injury. These pieces of information were obtained from participants’ medical history and a psychiatric interview. The study was approved by the Ethics Committee of Kermanshah University of Medical Science, and all subjects signed a written informed consent before participating in this study. All MRI images were visually checked by a radiologist to rule out any gross brain abnormality. After excluding 1 patient with hydrocephalus, 2 patients with comorbid periodic leg movements during sleep, 5 patients with mild obstructive sleep apnea, 2 patients with brain tumors, 2 ID patients and 4 HC individuals due to excessive head motions (translation > 1.5 mm and rotation > 1.5 degree), the analysis was performed based on 34 ID patients (15 PDI and 19 PPI) and 48 HCs.

### 2.2. Clinical Examination of ID Subtypes

ID patients were interviewed by a well-trained psychiatrist and sleep specialist (H.K.) according to the third version of ICSD diagnostic criteria. ID patients who were addicted to using any hypnotic medication, such as benzodiazepines, were excluded from the study. To diagnose ID subtypes, overnight polysomnography (PSG) using SOMNOscreenTM plus model (Somnomedics, Germany) was performed for all patients. The patients arrived at the Sleep Disorders Research Center at 9 p.m. and completed the demographic and PSQI questionnaires before polysomnography examination. PSG measurements were performed at least 7 h based on the routine sleeping habits of patients. Sleeping room conditions were standardized based on the international protocols of sleep labs [18]. Diagnosis of the ID subtypes was based on ICSD-2 definitions. Still, they all met criteria for “chronic ID” criteria based on ICSD-3, which consisted of subjective reports of disability to initiate and/or to maintain sleep (while there is an adequate opportunity to sleep) at least three times per week lasting for at least three months with associated daytime impairment. The insomnia symptoms were not due to substance abuse and other psychiatric comorbidities or other sleep disorders. PDI diagnosis was made through two criteria: i) discrepancy between objective and subjective reports, at least 1 h difference for total sleep time (TST) or 15% for sleep efficiency (SE), in a way that was contrary to a subjective report, objective sleep measures show near-normal sleep pattern; and ii) objective insomnia symptoms like TST more than 6 h and 30 min plus SE more than 85% [5,19]. Diagnostic criteria for PPI were based on subjective insomnia symptoms, and objective measures without any discrepancies between subjective and objective reports (i.e., SE less than 85% as well as TST less than 6.5 h) [18].

### 2.3. MRI and Resting-State fMRI Image Acquisition

Neuroimaging scans were conducted on subjects who stopped taking any hypnotic medication for at least one week to reduce the effects of medications on brain functions. Images were obtained through a 1.5 T scanner (Siemens Magnetom Avanto scanner) with an 8-channel head coil. We asked participants to stay still and awake, and additionally, to move as little as possible during the scan. Foam pads were used to reduce head motion artefacts. Anatomical T1 images were acquired by an MPRAGE sequence (TR = 1950 ms, TE = 3.1 ms, flip angle = 15°, FOV = 256 × 256 mm^2^, voxel size = 1 × 1 × 1 mm^3^, 176 sagittal slices). Functional images were collected from an EPI sequence (TR = 3 s, TE = 49 ms, flip angle = 15°, FOV = 64 × 64 mm^2^, voxel size = 3 × 3.5 × 3.5 mm^3^, time points = 200, 38 sagittal slices).

### 2.4. Preprocessing and Feature Extraction

To control any distortion and artefacts in the images, structural MRI images were carefully checked visually. To calculate structural measures, i.e., GMV and cortical thickness, we ran the FreeSurfer analysis (v.7.1.1 automatic pipeline) on the brainlife server (https://brainlife.io/). The pipeline automatically applies normalization to MNI space, bias field correction, brain extraction, segmentation, and surface reconstruction on sMRI. Finally, to have reasonable dimensions of the structural and functional measures [20,21], we extracted averaged cortical thickness and GMV features of 100 cortical (based on the Schaefer parcellation) and 36 subcortical parcels (based on the Brainnetome parcellation) [21,22,23].

Afterward, we performed preprocessing steps for functional images using the SPM toolbox DPABI5.2 [24]. The first 10 volumes were discarded to avoid magnetization instability. Pre-processing steps included slice timing correction, spatial realignment, segmentation, normalization to MNI space (2 × 2 × 2 mm^3^), smoothing with a 4 mm full-width-half-maximum (FWHM) Gaussian kernel, regressing out nuisance covariates (i.e., Friston-24 head motion parameters, white matter, and cerebrospinal fluid signal), detrending time series, and finally band pass filtering (0.01–0.10 Hz). Subsequently, for the 136 cortical and subcortical parcels, we calculated averaged ALFF, DC, and ReHo. Of note, Filtering was performed after the ALFF extraction, and additionally, conducted the smoothing after ReHo and DC calculation to prevent increasing regional similarity [25,26,27]. Moreover, ALFF was computed in the range 0.01–0.10 Hz, ReHo was computed based on a cluster of 27 voxels, and the correlation threshold was set to 0.25 for DC. Finally, to create our multimodal dataset we juxtaposed the extracted unimodal features (GMV, cortical thickness, ALFF, DC, ReHo) for each subject.

### 2.5. Statistical Analyses

We conducted chi-square tests to assess potential gender differences between the three groups and educational level differences between the two ID subtypes. Several One-Way Analysis of Variance (ANOVA) tests with Bonferroni correction were performed to test age, PSQI, intracranial volume, and head motion differences between groups. Moreover, we performed a *t*-test to compare SE and TST between PDI and PPI patients.

### 2.6. Classification

Before performing the principal component analysis (PCA) for dimension reduction, we performed normalization (L2 norm) since the features differed in variance and PCA is sensitive to variance [28]. Moreover, to classify our three groups (HC, PDI, PPI), we used the new support vector machine classification method (NuSVC) to separate groups, as applied previously due to its ability and precision [21,29]. NuSVC is a class of support vector algorithms that effectively control the number of support vectors [30]. The training was carried out in a grid search fashion for optimizing hyperparameters and choosing the best PCA component/components (Figure S1). Due to the number of subjects, the validation step was performed by leave-one-out (LOO) cross-validation. Of note, train and test data were carefully split to prevent data leakage. In the following, we conducted permutation tests with 2000 iterations to examine the classification reliability level [31]. In each iteration, we randomly assigned class labels to each sample and trained the ML model on the newly assigned samples to obtain a new accuracy (permutation accuracy). We calculated the *p*-value of classification accuracy as the proportion of permutation accuracies higher than the accuracy obtained using real samples. Finally, important features were selected by analyzing each feature’s amplitude of the eigenvectors, with features with higher amplitude indicating more power in classifications.

## 3. Results

**Figure 1:**
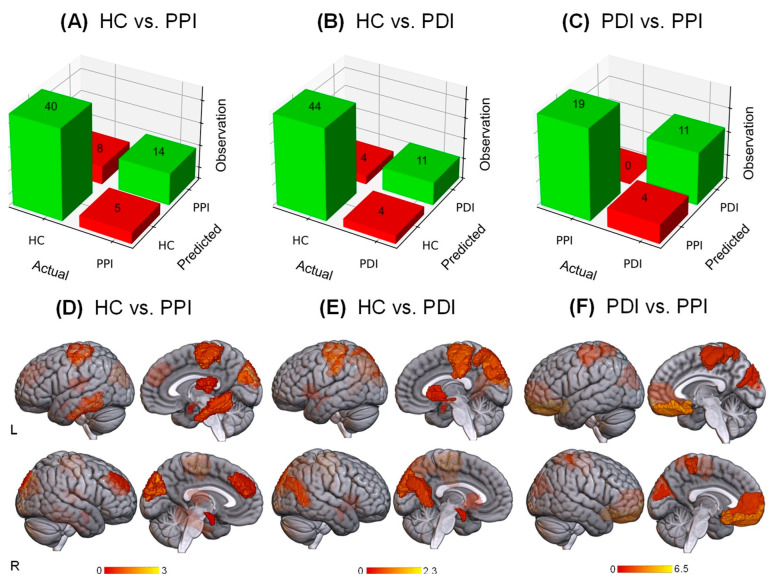
Multimodal confusion matrix (green color: correctly classified; red color: incorrectly classified) of HC vs. PPI classification shows 40 HCs and 14 PPIs were correctly classified (**A**). Confusion matrix of HC vs. PDI classification shows 44 HCs and 11 PDs were correctly classified (**B**). The confusion matrix of PDI vs. PPI classification shows 12 PDIs and 17 PPIs were correctly classified (**C**). The most important features (parcels) in each classification were illustrated: (**D**) HC vs. PPI, (**E**) HC vs. PDI, and (**F**) PDI vs. PPI. Important features were selected by analyzing each feature’s amplitude of the eigenvectors. The color bar indicates percent values of the amplitude of eigenvector for each feature. Cortical parcels were extracted by the Schaefer brain atlas and subcortical ones were extracted based on the Brainnetome atlas. HC: healthy control, PDI: paradoxical insomnia, PPI: psychophysiological insomnia.

Overall, 48 HC subjects were included (24 male, mean + SD age: 40.4 ± 12.7 years), as were 34 ID patients, 15 PDI (11 male, age: 42.3 ± 12.2 years) and 19 PPI (11 male, age: 44.6 ± 11.1 years). The demographic characteristics of all subjects and statistical comparison results are presented in Table S1. Moreover, classification results are as follows: (a) classification accuracy and ROC scores based on the first component were 81% and 79%, respectively, for HC vs. PPI with 89% sensitivity and 64% specificity (8 HCs and 5 PPIs were misclassified), (b) classification accuracy and ROC scores based on the first component were 87% and 82%, respectively, with 92% sensitivity and 73% specificity for HC vs. PDI (4 HCs and 4 PDIs were misclassified), and (c) classification accuracy and ROC scores based on the second component were 88% and 87%, respectively, with 82% sensitivity and 100% specificity for PDI vs. PPI (4 PDIs were misclassified). *p*-value outcomes of permutation tests for multimodal classifiers were less than 0.001 (Figure S2, Figure 1A–C, Table 1). One can see unimodal results in Table 1.

Analyzing the amplitude of eigenvector of corresponding PCA components clarified the features that had the most important roles in the multimodal classifications. The results are as follows: (a) GMV alterations in both right and left precentral gyrus, left inferior temporal gyrus, right paracingulate gyrus, left insula, and right amygdala between HC and PPI groups; (b) GMV abnormalities in the left precentral gyrus, left postcentral gyrus, left and right lateral occipital cortex, postcentral gyrus, left caudate, left putamen, and right amygdala for HC vs. PDI; and (c) GMV alterations in the left of the paracingulate gyrus and superior parietal lobule, and the right precentral gyrus between PDI and PPI groups (Figure 1E–G, Table S2).

## 4. Discussion

ID is the most prevalent sleep disorder with a wide range of mental health consequences. Hence, due to the heterogeneity and complex nature of this disorder, classifying its subtypes requires sophisticated computational methods, such as ML, which is rapidly expanding in the field of neuroimaging. Furthermore, precision psychiatry aims to bridge the gap between research discoveries and clinical applications. With this approach, we can gain a better understanding of brain dysfunction and optimal treatment of ID at the individual level. Our data-derived classification approach based on whole-brain structural and functional measures separated two ID subtypes, PPI and PDI, from each other and the HC group. Interestingly, we found that the classification based on multimodal neuroimaging data was partially superior to classifications based on unimodal data and mainly driven by structural data. We observed preliminary evidence that the ID subtypes could be separated using multimodal brain measures to tackle non-linearity and complex relations between groups. We believe that our study fulfills the criteria of a preliminary study, which searches for initial evidence that using multimodal imaging is helpful in separating individual patients with different subtypes of ID.

ICSD-3 discarded conventional subtypes of insomnia because of uncertainty about the reliability and validity of diagnostic tools and similar treatments for various subtypes. However, several studies suggested that ID is a heterogeneous disorder, and each ID subtype has a distinct clinical presentation and treatment response [7,11]. Moreover, this study is aligned with recent studies based on neuroimaging, PSG, and behavioral measures, suggesting that ID is not a unified entity. Moreover, a bottom-up classification of ID should be reconsidered in the field [5,7,20,32]. Previously, we assessed subcortical brain alterations in PDI and PPI and observed abnormality in the shape of the caudate, putamen, and nucleus accumbens in PDI and shape abnormality in the thalamus, amygdala, and hippocampus in PPI, suggesting a differential role of subcortical brain structures in the pathophysiology of two main subtypes of ID [5]. Furthermore, based on fMRI tasks, Lee and colleagues separated 19 PPI from 21 HCs with 80% accuracy. They applied PCA for dimension reduction and support vector machines for separation [20]. Moreover, using Random Forest on PSG data, Andrillon and colleagues separated PDI and non-PDI patients from each other and HCs. The authors demonstrated that the two ID subtypes could not be separated accurately (Cohen’s k = 0.004) if hypnogram indexes (e.g., TST, sleep onset latency, and duration of each sleep stage) were excluded [32].

Our classification based on a multimodal approach showed partially better classification accuracy compared with unimodal ones. Unimodal accuracies were different across HC vs. PPI, HC vs. PDI, and PDI vs. PPI classifications. These variations in classification results of the unimodal measures are probably due to each measure, representing an aspect of pathologic changes of the ID subtypes. Unimodal classification results show that for a specific classification such as PDI vs. PPI, specific unimodal imaging (e.g., GMV and DC) could get a comparable result to multimodal ones. Thus, the multimodal measure has been made up of combined unimodal measures and has the ability to boost differences between unimodal accuracies.

The main aim of this study is to provide preliminary evidence using advanced ML methods based on multimodal neuroimaging data to differentiate ID subtypes. However, several limitations should be considered while interpreting our results. First, due to restrictions during the COVID-19 pandemic, we were unable to recruit a larger sample size in each subtype group, particularly PDI patients. Of note, a larger sample size is required to consider variability between subjects. Larger sample sizes contain more data, which enables ML-based classification to capture more information during the training phase to make decisions about unseen data. Second, LOO cross-validation introduces high variance in the reported results of the classification and uses a set of highly similar training sets, which increases the variability of LOO results. Finally, out-of-sample validation is necessary to evaluate the generalizability and reproducibility of our classification results in independent samples.

## 5. Conclusions

This preliminary study provides evidence that structural and functional brain measures can help to distinguish two common subtypes of ID from each other and healthy subjects. Moreover, we observed that the multimodal brain measure is better than the unimodal brain measure to separate ID subtypes. Future studies using larger sample sizes, such as the ENIGMA-Sleep consortium [33], should further investigate neurobiological mechanisms underlining ID subtypes and their comorbid conditions, such as depression and anxiety, to optimize and develop new personalized therapeutic approaches. 

## Figures and Tables

**Table 1:** Outcomes of unimodal and multimodal classifications.

Measures	Validation	HC vs. PPI	HC vs. PDI	PDI vs. PPI
Multimodal	Accuracy %	81	87	88
	ROC score %	79	82	87
	Sensitivity %	89	92	82
	Specificity %	64	73	100
	*p*-value	<0.001	<0.001	<0.001
Cortical Thickness	**Accuracy %**	**78**	**67**	**79**
	ROC score %	72	74	81
	Sensitivity %	84	93	93
	Specificity %	61	59	70
	*p*-value	<0.001	0.125	0.01
Whole Brain GMV	**Accuracy %**	**78**	**87**	**85**
	ROC score %	75	82	85
	Sensitivity %	87	92	85
	Specificity %	59	73	86
	*p*-value	0.003	<0.001	<0.001
Whole Brain ALFF	**Accuracy %**	**85**	**79**	**71**
	ROC score %	82	66	70
	Sensitivity %	94	83	74
	Specificity %	74	60	67
	*p*-value	<0.001	0.004	0.014
Whole Brain DC	**Accuracy %**	**72**	**70**	**82**
	ROC score %	67	69	82
	Sensitivity %	82	87	84
	Specificity %	50	42	80
	*p*-value	0.004	0.012	<0.001
Whole Brain ReHo	**Accuracy %**	**70**	**83**	**76**
	ROC score %	78	84	76
	Sensitivity %	83	95	79
	Specificity %	63	59	73
	*p*-value	0.006	0.016	0.005

HC: healthy control, PPI: psychophysiological insomnia, PDI: paradoxical insomnia, ROC: the area under the receiver operating characteristic, GMV: grey matter volume, ALFF: amplitude of low-frequency fluctuation, DC: degree centrality, ReHo: regional homogeneity.

## Data Availability

The dataset presented in the study is available upon request from the corresponding author upon reasonable request.

## Supplementary Materials

The following supporting information can be downloaded at: https://www.mdpi.com/article/10.3390/brainsci13040672/s1, Figure S1: Multi-modal PCA eigenvalues; Figure S2: Permutation test; Table S1: Demographic and clinical variables of the participants; Table S2: The most important brain features in each classification.
