# Supplementary material for: Discriminating Paradoxical and Psychophysiological Insomnia Based on Structural and Functional Brain Images: A Preliminary Machine Learning Study
Source: Brain Sci. 2023 Apr 17;13(4):672. doi: 10.3390/brainsci13040672 (PMC10136511; doi:10.3390/brainsci13040672)
Supplement: Supplementary file 1 [file brainsci-13-00672-s001.zip › brainsci-2306807-supplementary.pdf]

Supplementary Material

Discriminating paradoxical and psychophysiological insomnia based on structural and functional brain images: a preliminary machine learning study

Figures:

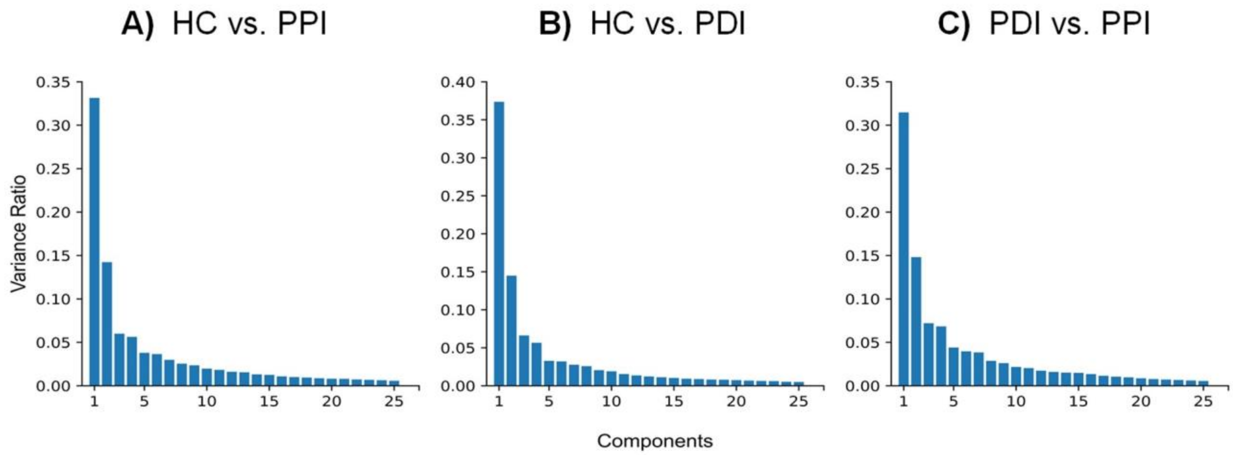

Figure S1 | Multi-modal PCA eigenvalues show the first and second components contain more detail than others. HC vs. PPI classification (A). HC vs. PDI classification (B). PDI vs. PPI classification (C). HC: healthy control, PDI: paradoxical insomnia, PPI: psychophysiological insomnia, PCA: principle component analysis.

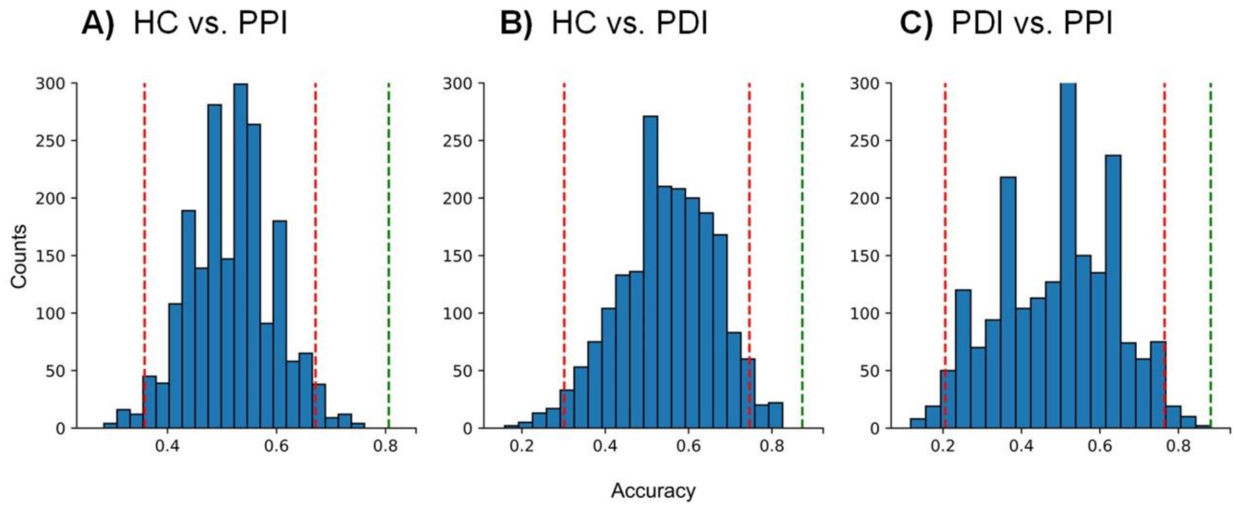

Figure S2| Permutation tests of multimodal classification highlight p-value < 0.001 for HC vs. PPI classification (A), HC vs. PDI classification (B), and PDI vs. PPI classification (C). Red lines show 95%

confidence interval and green lines indicate real accuracies. Real accuracies are 81, 87, and 89% respectively for a, b, and c. HC: healthy control, PDI: paradoxical insomnia, PPI: psychophysiological insomnia.

Table S1 | Demographic and clinical variables of the participants.

Variables	HC (48)	PPI (19)	PDI (15)	P-value (HC vs. PPI)	P-value (HC vs. PDI)	P-value (PDI vs. PPI)
**Gender (M, F)**	24, 24	11, 8	11, 4	0.560	0.112	0.350
**Age (mean± SD)**	40.35 ± 12.67	44.58 ± 11.12	42.33 ± 12.23	0.622	1.000	1.000
**Head motion (mean± SD)**	0.091 ± 0.052	0.078 ± 0.033	0.088 ± 0.052	0.841	1.000	1.000
**TIV (mean± SD)**	1.59 ± 0.144	1.53 ± 0.167	1.56 ± 0.211	0.542	1.000	1.000
**PSQI (mean± SD)**	3.33 ± 1.79	17.16 ± 3.39	16.47 ± 2.03	< 0.001	< 0.001	1.000
**TST (hour) (mean± SD)**	-	4.02 ± 1.91	6.63 ± 0.56	-	-	< 0.001
**SE (%) (mean± SD)**	-	52.66 ± 25.28	86.46 ± 6.90	-	-	< 0.001
**Educational level (1, 2, 3)**	-	5, 6, 6	6, 2, 5	-	-	0.432

Chi-square test results did not show any significant gender differences between the three classes and also did not show any significant educational level differences between two insomnia disorder subtypes. Educational level were arranged from 1 as primary school, 2 high school, and 3 as university levels. One-way ANOVA results (Bonferroni correction) show no age, head motion, and TIV differences between the classes. One-way ANOVA results (Bonferroni correction) show PSQI differences for control vs. psychophysiological and control vs. paradoxical but not for paradoxical vs. psychophysiological patients. Independent T-test shows significant total sleep time and sleep efficiency differences between paradoxical and psychophysiological patients. HC: healthy control, PPI: psychophysiological insomnia, PDI: paradoxical insomnia, TIV: total intracranial volume, PSQI: total Pittsburg Sleep Quality Index score, TST: total sleep time. SE: sleep efficiency.

Table S2 | The most important brain features in each classification.

Region	L/R	MNI coordinate			Magnitude of eigenvectors %
**a) HC vs. PPI**					
Precentral gyrus	L	-40	-22	58	3
Precentral gyrus	R	46	-12	48	2.9
Inferior temporal gyrus superior	L	-58	-32	-22	2.2
Paracingulate gyrus	R	6	28	30	1.8
Insula	L	-36	-22	16	1.5
Amygdala	R	123	97	193	0.6
**b) HC vs. PDI**					
Precentral gyrus	L	-40	-22	58	2.2
Postcentral gyrus	L	-58	-24	38	1.6
Lateral occipital cortex superior	L	-24	-68	50	1.6
Lateral occipital cortex superior	R	36	-82	16	1.4
Postcentral gyrus	R	50	-24	42	1.4
Caudate	L	0	76	211	0.6
Putamen	L	70	26	53	0.43
Amygdala	R	123	97	193	0.43
**c) PDI vs. PPI**					
Frontal orbital cortex	L	-14	32	-20	6.5
Frontal pole	R	12	35	-20	5.8
Postcentral gyrus	R	30	-37	64	2.4
Superior parietal lobule	L	-22	-51	66	2.2
Paracingulate gyrus	R	7	48	1	2.2
Precentral gyrus	L	-39	-23	59	2.1
Cuneal cortex	L	-6	-82	26	1.6

HC: healthy control, PPI: psychophysiological insomnia, PDI: paradoxical insomnia.
